# Visual Online Control of Goal-Directed Aiming Movements in Children

**DOI:** 10.3389/fpsyg.2016.00989

**Published:** 2016-07-05

**Authors:** Isabelle Mackrous, Luc Proteau

**Affiliations:** Département de Kinésiologie, Université de Montréal, MontréalQC, Canada

**Keywords:** online motor control, manual aiming, automatic corrections, internal model, visual feedback, cursor jump

## Abstract

The present study investigated whether the initial impulse of goal-directed movements was visually monitored by 5- to 12-years-old children (*n* = 36) in a manner similar to adults (*n* = 12). The participants moved a cursor toward a fixed target. In some trials, the cursor was unpredictably translated by 20 mm following movement initiation. The results showed that even the youngest children visually monitor the initial impulse of goal-directed movements. This monitoring and the error correction process that it triggers seem automatic because it occurs even when the cursor jump is not consciously detected. Finally, it appears that this process does not fully mature before late childhood, which suggests that a putative dedicated channel for processing visual hand information develops during childhood.

## Introduction

Goal-directed movements elicit a series of processes to identify a target and its location and to transform this information into appropriate motor commands. With practice, movement planning and execution processes become more accurate. However, the intrinsic variability present in all human processes and the high level of accuracy that is required in many of our daily activities require that the central nervous system (CNS) closely monitors our movements to quickly update movement planning and amend movement execution (Desmurget and Grafton, 2000; Franklin and Wolpert, 2008; Vesia et al., 2008; Franklin et al., 2012). Specifically, it is thought that an efference copy of the motor commands is sent to a forward model that anticipates their sensorimotor consequences, predicts the movement endpoint, and when necessary, issues corrective motor commands. The forward model is updated/fine-tuned during movement execution by incoming proprioceptive and visual inputs (for a review, see, Desmurget and Grafton, 2000; Shadmehr et al., 2010). In this study, we investigated whether—and eventually how—children in different age groups amend a movement that is not progressing as planned.

Many researchers have used a perturbation paradigm to investigate error detection and correction processes. If the perturbations are infrequent and unexpected, it is thought that participants plan their movements as if no perturbation would occur. Therefore, participants in the perturbed trials must correct the movement that they have planned and initiated to counteract the perturbation, and this correction provides insight into error detection and correction processes. For instance, in many studies, adult participants performed a video aimed movement for which they used a computer mouse to move a cursor on a computer screen toward a fixed target displayed on the screen. In some trials, the position of the target (Day and Lyon, 2000; Sarlegna et al., 2003; Gritsenko and Kalaska, 2010; Franklin et al., 2012; Hyde and Wilson, 2013; Wilson and Hyde, 2013) or of the cursor (Sarlegna et al., 2003, 2004; Saunders and Knill, 2003, 2004; Franklin and Wolpert, 2008; Proteau et al., 2009; Veyrat-Masson et al., 2010; Brière and Proteau, 2011) was translated by 10–40 mm immediately prior to, during, or subsequent to initiation of the movement. Similar correction latencies were found for a target- or a cursor jump, which ranged between 117 and 160 ms following the perturbation (Prablanc and Martin, 1992; Brenner and Smeets, 2003; Saunders and Knill, 2003, 2004; Franklin and Wolpert, 2008), suggesting that the corrections for these two types of perturbations share similar processes. Moreover, the results of target- and cursor jump studies show that even when participants did not consciously detect the jump, they quickly and accurately corrected for it (Goodale et al., 1986; Brière and Proteau, 2011, 2016). Finally, when the participants were instructed to move their hand in the direction of the target- (Day and Lyon, 2000) or the cursor jump (Franklin and Wolpert, 2008), they failed to refrain from performing a corrective response in the direction opposite to the jump. Together, these results suggest that the forward model initiates an automatic movement correction process in response to both types of perturbations. Observing that a correction occurred for the first perturbed trial (Brière and Proteau, 2011, 2016) and that the latency of a correction was not affected by the location (Saunders and Knill, 2003, 2004; Brière and Proteau, 2011) or size (Veyrat-Masson et al., 2010) of the cursor jump suggest a continuous monitoring of cursor displacement as movement unfolded in adults.

The results of two recent studies suggest that these automatic error correction processes might not be fully developed in young children (Hyde and Wilson, 2013; Wilson and Hyde, 2013). These authors used a target jump paradigm and noted that participants as young as 5–6 years redirected most of their movements toward the new target location. For all age groups, movement time was longer for the jump trials than for the control trials. However, this increase in movement time was significantly longer for 5- to 6-years-old children than for older children and adults. In addition, the latency of a correction for a target jump significantly decreased from 5- to 6-years-old children, to older children, and to adults. Finally, the 5- to 6-years-old children spent significantly more time than older children or adults reaching the target once a correction was initiated, but nonetheless missed the target significantly more often than the other three age groups. It had already been shown that young children are spatially less accurate and more variable than older children and adults (Bard et al., 1990; Lhuisset and Proteau, 2002, 2004). For the first time, the results reported above (Hyde and Wilson, 2013; Wilson and Hyde, 2013) suggest that this might at least partially results from the immaturity of the so-called automatic error detection and movement correction processes put into play when a sudden change in target location occurs. However, regardless of the many similarities reported above between corrections for target- and cursor jump perturbations, recent findings suggest that corrections for a cursor jump might be of an even more automatic or “reflex-like” nature (Franklin and Wolpert, 2008) than corrections for a target jump. Our goal was to determine whether these more fundamental error detection and correction processes are mature in children.

Specifically, Reichenbach et al. (2014) used a video bimanual reaching task in which the participants aimed one cursor controlled by their right hand toward a target located on their right and aimed a second cursor controlled by their left hand toward a second target located on their left. During one block of trials, either one of the two targets or one of the two cursors could jump. The results showed that attracting the participant’s attention to the side of a target jump resulted in faster and more accurate corrections, whereas this was not the case for the corrections performed for a cursor jump. In another experiment, participants had to intercept a moving target with a cursor controlled by their right hand in the presence of up to four visual distractors. As in the previous task, either the target or the cursor could jump across different blocks of trials. The results showed that the corrections for the cursor jump were less negatively affected by the presence of distractors than the corrections for a target jump. Taken together, these results suggest that although the processing of a visual target and of hand information are both automatic and involuntary, the processing of visual hand information appears to occur through a dedicated channel that is not influenced by the allocation of visual attention. In turn, this suggests that the detection and correction of errors resulting from one’s actions are fundamental processes for the control of goal-directed movements (Reichenbach et al., 2014). If so, it might be hypothesized that even young children would be able to correct their movement for a cursor jump as efficiently and as quickly as older children and adults. Our specific goal was to test this hypothesis.

## Materials and Methods

### Participants

Thirty-six children (*n* = 12 for each of the 6–7, 8–9, and 10–12 years-old groups) and 12 adults between the ages of 20 and 30 years participated in this experiment. All child participants were recruited from a summer camp at the Sports Center of a large Montreal university. All children lived in the demographically diverse Greater Montreal area and received a toy for their participation (retail value = $10 CDN). Consent forms were distributed to the parents 1 week prior to data collection. Only children who returned a signed consent form participated in the study. In addition, each child provided verbal assent prior to participating in the experiment. The adult participants were undergraduate students in the Kinesiology Department who provided a signed consent form. These participants were paid $10 CDN for their participation. The participants were right-handed, and they (or their parent) reported normal or corrected-to-normal vision. The Health Sciences Ethics Committee of the authors’ institution approved this study.

### Task and Apparatus

**FIGURE 1:**
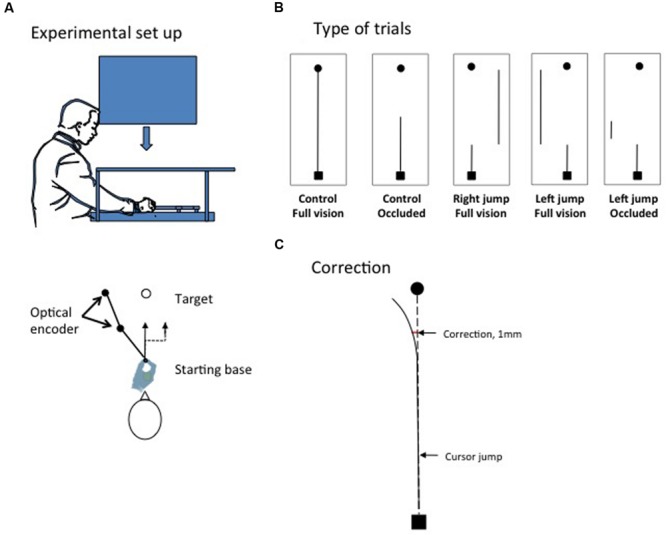
**(A)** The experimental set-up. **(B)** The different types of trials. The black dot indicates the target; the black square indicates the starting base. The thin black line indicates the trajectory of the cursor. The cursor could be seen from start to finish (control full vision, right and left jump full vision) or masked during movement execution (control no vision and left jump occluded). For some trials, the position of cursor was unexpectedly translated by 20 mm soon after movement initiation (target jump conditions). **(C)** The initiation of a correction for a cursor jump was detected when, in relation to control trials, the stylus moved by the participant deviated by 1 mm in the direction opposite to the cursor jump (see Materials and Methods).

The task involved moving a computer-mouse-like device from a fixed starting position close to the body toward a target located 150 mm away from the body. The apparatus consisted of a table, computer screen, headrest, mirror, and a two-degree-of-freedom manipulandum (**Figure 1A**). The participants were seated in front of the table. A CRT computer screen (Mitsubishi, Color Pro Diamond, 37 inches, refresh rate 60 Hz) was mounted on a ceiling support positioned directly over the table and was oriented parallel to the surface of the table. The table’s image was reflected in a mirror placed directly beneath and parallel to the tabletop. The mirror was located at the midpoint between the computer screen and tabletop (37 cm apart) and permitted free displacement of the manipulandum on the tabletop. The information presented on the computer screen was thus reflected in the mirror and was visible to the participant. The participant’s chair height was adjusted such that their forehead could rest comfortably on a headrest. The headrest was aligned with the lateral center of the computer screen and was used to standardize the information displayed on the computer screen for all participants.

The tabletop was covered by a piece of Plexiglas, over which the starting base and manipulandum were fixed. The starting base consisted of a thin strip of Plexiglas glued to the tabletop. This strip was parallel to the leading edge of the table and had a small indentation on its distal face, and this indentation was aligned with the headrest and the participant’s midline and served as the starting base for the stylus (see below). The indentation made it easy for the participant to position the stylus at the beginning of each trial.

The manipulandum consisted of two pieces of rigid Plexiglas (43 cm) joined at one end by an axle. One free end of the manipulandum was fitted with a second axle encased in a stationary base. The other free end of the manipulandum was fitted with a small vertical shaft (length: 3 cm; radius: 1 cm), i.e., the stylus, which could be gripped by the participant. Each axle of the manipulandum was fitted with a 13-bit optical shaft encoder (U.S. Digital, model S2-2048, sampled at 500 Hz, angular accuracy of 0.0439°), which enabled us to track the displacement of the stylus online and display it in a 1:1 ratio on the computer screen. Moving the stylus away from the body in the frontal and sagittal planes resulted in an identical displacement of the cursor on the computer screen. The bottom of the stylus and bottom of the optical encoder were located at the junction of the two arms of the manipulandum and covered with a thin piece of Plexiglas. By lubricating the working surface at the beginning of each experimental session, the stylus displacement was nearly frictionless.

### Procedure

The participants attempted to stop the cursor (red; 3 mm in diameter) on a black target (6 mm in diameter). The target was 150 mm in front of the starting base (i.e., in line with the participant’s midline). The participants were asked to use their right hand to initiate their movement at will following the presentation of the target (i.e., not a reaction time task) and to perform smooth and continuous movements (i.e., not a stop-and-go strategy). The participants were also required to complete their movements within a time frame ranging from 480 to 620 ms (500 ms ± 12%). When the movements were completed outside this time frame, the experimenter reminded the participant of the target movement time. A movement time bandwidth (Saunders and Knill, 2003, 2004; Proteau et al., 2009) reduces the possibility of different speed-accuracy trade-offs between the different experimental conditions (Fitts, 1954). In addition, in this experiment, it enabled us to determine whether the automatic control processes proposed for the correction of a cursor jump in adults also take place at no temporal cost in children.

At the beginning of each trial, all participants could see the cursor resting on the starting base. Once the stylus was stabilized on the starting base for 500 ms, the target was presented on the screen. Movement initiation was detected during data acquisition when the cursor was moved 1 mm, and movement completion was detected when the cursor did not move more than 2 mm in a time frame of 100 ms. This procedure permitted us to determine whether cursor-jump corrections (see below) occurred during the initial impulse of movement (i.e., prior to late corrections that occur while the cursor approaches the target, Mackrous and Proteau, 2007; Grierson and Elliott, 2009). When movement completion was detected, the position of the cursor endpoint and the target were visible for 500 ms. Therefore, the same information concerning the movement endpoint was available in all the experimental conditions described below.

The participants initially participated in a familiarization phase that consisted of 10 control trials completed in normal vision (see below). During the familiarization phase, special attention was given to children ensuring that they performed the task as required. Specifically, the experimenter made a demonstration of the single motion movement to be produced and of the multiple sub-movements approach to be avoided. Then, on every familiarization trials, the experimenter gave verbal feedback to the children regarding the fluidity (i.e., one or two sub-movements) and the accuracy of the movement (*i.e., you hit/missed the target*) as well as on movement time *(i.e., correct, too fast or to slow*). The experimental session begun when the children fully understood the constraints of the task, which was normally achieved within the 10 familiarization trials. This phase was followed by 110 experimental trials, which took approximately 30 min to complete for the younger children. Details concerning the different types of control and cursor-jump trials in this experimental phase are summarized in **Figure 1B**, which shows that two types of control trials were performed. For 80 control trials, the cursor was visible for the entire duration of the movement (control full vision), whereas for 15 trials, the cursor was occluded after it had moved 70 mm (control occluded). A cursor jump occurred in 15 trials. Specifically, after the cursor had moved 35 mm, it was translated 20 mm perpendicularly to the right or left of the hand (stylus) position. The cursor was either visible for the entire movement duration (right jump full vision and left jump full vision; five trials for each condition) or occluded for the remaining five trials after the cursor had moved 70 mm (35 mm after a left cursor jump had occurred, the cursor was occluded). The control-occluded trials and cursor-jump trials were randomly presented with the restriction that one trial of each type occurred once within each successive block of 22 trials. Note that, verbal feedback concerning movement time was given to the children if they showed a tendency to perform movements too quickly or too slowly.

### Data Reduction

The tangential displacement data of the stylus over time were first smoothed using a second order recursive Butterworth filter with a cut-off frequency of 10 Hz. The filtered data were numerically differentiated once using a central finite technique to obtain the velocity profile of the aiming movement, a second time to obtain the acceleration profile, and a third time to obtain a jerk profile. We determined the end of the movement’s primary impulse from the kinematic profiles (Meyer et al., 1988). This occurred when one of the following events was detected on the kinematic profiles: (a) movement velocity falling below 20 mm/s, (b) movement reversal (velocity going from positive to negative), (c) movement lengthening (presence of a secondary movement impulse as indexed by the acceleration profile crossing the zero value for a second time) or (d) significant disruption in the deceleration profile as indexed by zero-crossing on the jerk profile. A secondary movement impulse was considered a discrete correction when its duration was at least 80 ms and its extent was at least 2 mm.

Movement initiation was detected when the stylus was moved 1 mm to provide quick feedback to the participant during data acquisition. However, movement initiation was defined as the moment at which the tangential velocity of the cursor reached 10 mm/s and was maintained above this value for at least 20 ms for the primary analyses. Visual inspection of the data revealed that movement was clearly underway once a velocity of 10 mm/s was reached. Movement endpoint was defined as the end of the movement’s primary impulse using the parsing algorithm defined above.

To evaluate whether the participants corrected for the perturbation during the initial movement impulse (hereafter called online correction), we determined the *frontal* and *sagittal* position of the hand relative to the center of the target at movement endpoint (Cartesian coordinates). In the frontal dimension, a positive value indicated that the hand ended to the right of the target, whereas a negative value indicated that the hand ended to the left of the target. A cursor-jump correction was expected to occur mainly in this dimension of the task. A full correction would result in the hand’s movement ending 20 mm to the right or left (for left and right cursor jumps, respectively) of the mean hand position for the control trials. In the sagittal dimension, a positive value indicated that the hand overshot the target, whereas a negative value indicated that the hand undershot the target. In addition, we also computed within-participant variability on both task dimensions (i.e., within-participant standard deviation of movement endpoint).

Performances on the three types of cursor-jump trials (i.e., right jump full vision, left jump full vision, and left jump occluded trials) were contrasted with those of randomly selected control trials although they did not immediately follow a cursor-jump trial. Although, the participants were instructed not to perform a discrete correction (i.e., stop-and-go), we observed that regardless of the trial type, as many as 50% of the trials with children showed a secondary movement impulse. These trials were not discarded from the analyses. However, for the children, we performed a series of supplemental analyses in which we contrasted the performances of trials showing or not showing a secondary corrective impulse.

We also determined the latency of the correction for a cursor jump. We used the hand frontal location data because the cursor jump and the expected correction primarily occurred on this axis. We computed the mean frontal location of the hand for the five types of trials defined above for each participant. Then, we computed the difference in the location of the stylus between the control full vision and the cursor jump trials every 20 ms. A correction for the cursor jump was detected when a cursor jump condition deviated from the control full vision condition by more than 1 mm in the direction opposite that of the cursor jump (see **Figure 1C**). The 1-mm criterion was chosen arbitrarily. Correction onset was also detected only when the change in direction continuously increased as movement unfolded and became significant to ensure that we did not obtain a false positive. This technique was used by Proteau et al. (2009), who reported latencies in the same range as previous reports (Brenner and Smeets, 2003; Saunders and Knill, 2003).

## Results

### Cursor-Jump Corrections

#### Frontal Dimension

To determine whether the participants corrected their movements in reaction to the cursor jump, the hand frontal bias and variability at the end of the movement’s initial impulse were investigated independently using an ANOVA that contrasted 4 age groups (children aged 6–7, 8–9, and 10–12 years and adults) × 5 types of trials (control full vision, control occluded, right jump full vision, left jump full vision, and left jump occluded) with repeated measurements on the last factor.

**FIGURE 2:**
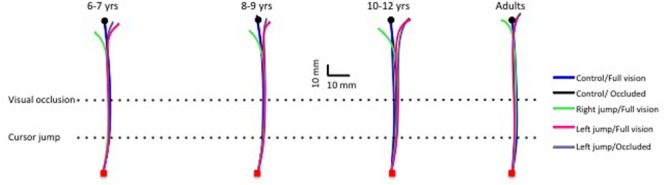
**Typical movement trajectories for control and cursor-jump trials for all age groups (one typical participant for each age group).** Note the correction for the cursor jump even for the youngest children group.

**FIGURE 3:**
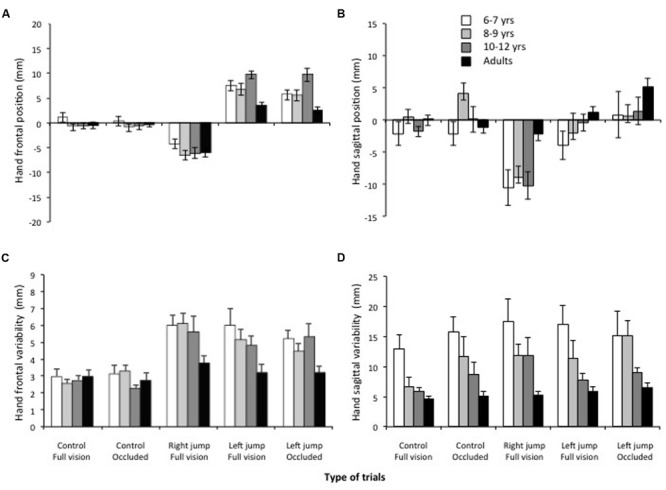
**Stylus (hand) frontal **(A)** and sagittal **(B)** position at the end of the movement initial impulse as a function of the age groups and the types of trials.** Stylus (hand) frontal **(C)** and sagittal **(D)** within-participant variability at the end of the movement initial impulse as a function of the age groups and the types of trials. Note the correction for the cursor jump in all age groups even when the cursor was occluded soon after the cursor jump (left jump occluded).

The ANOVA computed on the frontal endpoint constant error (i.e., frontal bias) revealed a significant age group x type of trial interaction, *F*(12,144) = 3.97, *p* < 0.001, η^2^ = 0.17. The breakdown of this interaction revealed that the hand frontal position did not differ across ages for the full vision and occluded control trials (**Figures 2** and **3A**), *F*(3,36) = 0.7 and 1.77, *p* = 0.10, respectively. Because no significant difference was observed between the age groups for the control trials, any difference in hand position for the cursor-jump trials should have resulted from a correction for this visual perturbation. **Figures 2** and **3A** show that the participant’s hand position significantly ended to the left and right of that of the control trials for the right- and left-cursor-jump trials, respectively. Thus, participants in all age groups modified their movement’s initial impulses in reaction to the cursor jump. The breakdown of the above-mentioned interaction further revealed that for the right cursor jump, the size of this correction (mean of 6.5 mm) did not differ significantly among the age groups, *F*(3,36) = 1.6, *p* = 0.21. However, for the left cursor jump in normal or occluded trials, the correction by the adults was significantly smaller than the correction by the children, *F*(3,36) = 5.45, and 7.66, *p* = 0.01. In addition, error corrections were smaller for the two younger groups of children than for the 10- to 12-years-old group (only significant for the occluded trials; means of 6.8 [6–7 and 8–9 years], 10.6 [10–12 years], and 3.3 mm adults]). No other effect was significant.

Finally, concerning the endpoint frontal variability (**Figure 3C**), the ANOVA revealed significant main effects of age group, *F*(3,) = 6.7, *p* < 0.005, η^2^ = 0.35, and trial type, *F*(4,144) = 15.8, *p* < 0.001, η^2^ = 0.29. All children showed larger endpoint frontal variability than the adults. Frontal variability was significantly smaller for the control than for cursor-jump trials. No significant difference was observed within these two sets of trials (all *p*s > 0.30).

#### Sagittal Dimension

Although the cursor jump did not require an adjustment of the planned movement amplitude, the movement amplitude had to be controlled for the hand to end on the target. The hand sagittal bias and variability data were analyzed identical to the endpoint frontal data.

Concerning a sagittal bias, the ANOVA indicated a significant main effect of trial type, *F*(4,144) = 21.06, *p* < 0.001, η^2^ = 0.37. **Figure 3B** shows that this effect revealed slight but significantly shorter movements when the cursor jumped to the right (8.2 mm) than for any other trial types, which did not significantly differ from one another (control occluded +0.5 mm; control vision: -0.8 mm; and left occluded: +1.7 mm). The ANOVA for sagittal endpoint variability (**Figure 3D**) revealed a significant main effect of trial type, *F*(4,144) = 2.8, *p* = 0.03, η^2^ = 0.07, thus indicating a significantly smaller variability for the control trials completed in normal vision (7.6 mm) than for any other trial type, which did not differ significantly from one another (mean of 11. 4 mm). There was also a significant main effect of age, *F*(3,36) = 7.4, *p* < 0.001, η^2^ = 0.37), which indicated that endpoint sagittal variability significantly decreased with age (from 16.3 to 5.4 mm).

### Movement Time

To determine whether the participants respected the movement time constraint, movement time and movement time variability were analyzed using an ANOVA contrasting the 4 age groups (children aged 6–7, 8–9, and 10–12 years and adults) × 5 types of trials (control full vision, control occluded, right jump full vision, left jump full vision, and left jump occluded).

**FIGURE 4:**
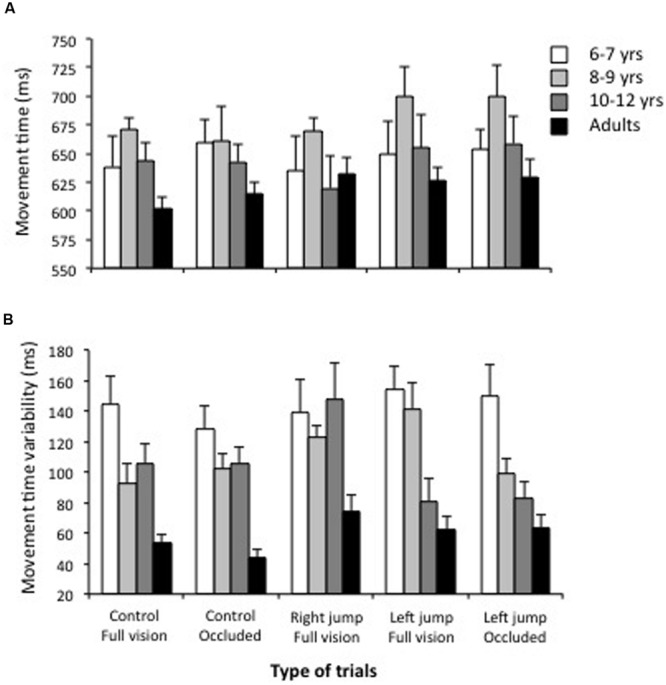
**Movement time **(A)** and movement time variability **(B)** as a function of the age group and the types of trial.** Note that children had greater variability in movement time than adults.

**Figure 4A** shows the mean movement time and indicates that all age groups had longer (30%) movement times than the mean prescribed movement time of 500 ms. The ANOVA indicated that the 8- to 9-years-old children had slightly longer movement times than the other children and adults, *F*(3,36) = 2.4, *p* = 0.08, η^2^ = 0.16. Movement times were slightly longer for the left-cursor-jump trials completed with or without vision of the cursor (670 and 673 ms, respectively) than for the remaining trial types that did not significantly differ from one another (range of 639 to 644 ms), *F*(4,144) = 3.14, *p* = 0.016, η^2^ = 0.26.

The ANOVA performed to analyze movement time variability indicated significant main effects for the age groups, *F*(3,36) 16.6, *p* < 0.001, η^2^ = 0.55 and trial types, *F*(4,144) = 2.84, *p* = 0.03, η^2^ = 0.06, as well as a significant age groups x trial types interaction, *F*(12,144) = 2.05, *p* = 0.03, η^2^ = 0.12. **Figure 4B** shows the overall movement time variability decreased with age. The breakdown of the interaction further revealed that the trial type did not significantly influence movement time variability for the youngest children and the adults, *F*(4,33) = 1.4 and 1.1, *p* > 0.20. Although, the trial type significantly modified the movement time variability for the groups of children aged 8–9 and 10–12 years, *F*(4,33) = 2.98 and 2.85, *p* < 0.05, respectively, the data did not show a consistent trend.

### Correction Latency

To determine whether the latency of the cursor-jump correction differed across age groups and trial types, the latency data were analyzed with an ANOVA contrasting the 4 age groups (children aged 6–7, 8–9, 10–12 years, and adults) × 3 types of perturbed trials (right jump full vision, left jump full vision, and left jump occluded) with repeated measurements on the last factor. Two participants from the 6- to 7-years-old group and 1 participant from the 8- to 9-years-old group were excluded from this analysis because there was no correction evidence for the left jump occluded trials (i.e., the mean trajectories of both perturbed and control trials overlapped). Therefore, correction latency could not be determined. The ANOVA did not indicate a significant main effect or interaction (*p*s > 0.19). Nonetheless, the data reported in **Table 1** suggest that the latency of a cursor-jump correction might slightly decrease with age: the 10- to 12-years-old and adults were 13% faster overall than the two younger groups of children (Hyde and Wilson, 2013; Wilson and Hyde, 2013).

**Table 1:** Mean latency (standard deviation) of the correction for the cursor jump (ms).

	Types of perturbed trials		
	Right jump Full vision	Left jump Full vision	Left jump No-vision
6–7 years-old	281 (31)	307 (33)	265 (25)
8–9 years-old	270 (29)	278 (26)	256 (22)
10–12 years-old	256 (19)	243 (10)	243 (13)
Adults	260 (17)	231 (17)	240 (10)

### A Descriptive Analysis of the Discrete Corrections Performed by Children in Reaction to a Cursor Jump

Although the participants were instructed not to execute discrete corrections, the children (but not the adults, <5%) had a large proportion of trials showing a secondary corrective impulse. **Table 2** presents a series of descriptive data characterizing these secondary corrective impulses. Because of the small proportion of cursor-jump trials in each subcategory, we opted not to compute any statistical analyses on the data presented in **Table 2**. On average, children performed discrete corrections in cursor-jump trials three times more than in control trials (45 vs. 13%, respectively). This result suggests that these discrete corrections were performed to counteract the cursor jump. This position is supported by the observation that for the cursor-jump trials with a secondary correction, the correction amplitude at the end of the secondary corrective impulse was more than twice as large than that at the end of the initial impulse movement. Notably, and finally, the secondary corrections observed in children, although, larger when the cursor was visible throughout movement execution, obviously occurred and were efficient when the cursor was occluded soon after the cursor jump. Therefore, the visual information concerning the position of the displaced cursor provided sufficient input to an internal model of limb kinematics to initiate a correction while visual feedback remained available and complemented this initial correction with a secondary correction after vision was withdrawn.

**Table 2:** Characteristics of secondary corrections in children.

	Type of trials				
	**Control**	**Control**	**Left-jump**	**Left-jump**	**Right-jump**
	**NV**	**TO**	**NV**	**occluded**	**NV**
	
	**Proportion of trials showing a secondary corrective impulse**				
	
6–7 years-old	20%	22%	55%	50%	36%
8–9 years-old	4%	6%	32%	22%	52%
10–12 years-old	20%	8%	62%	28%	66%
Adults	2%	0%	5%	4%	16%
	
	**Amplitude of the correction during the movement initial impulse; trials showing a secondary corrective impulse**				
	
6–7-years-old	2.5 mm	0.3 mm	4.3 mm	7.9 mm	-4.0 mm
8–9 years-old	-2.4 mm	2.2 mm	6.6 mm	7.3 mm	-5.9 mm
10–12 years-old	1.9 mm	2.5 mm	9.2 mm	9.8 mm	-4.0 mm
	
	**Amplitude of the correction at the end of the secondary impulse**				
	
6–7-years old	1.3 mm	-1.0 mm	15.8 mm	10.8 mm	-14.6 mm
8–9-years old	-2.4 mm	0.7 mm	17.4 mm	11.2 mm	-15.4 mm
10–12 years-old	1.1 mm	1.0 mm	15.8 mm	17.8 mm	-14.7 mm

*NV, normal vision.*

## Discussion

The objective of the present study was to determine whether early online visual control of goal-directed movement relied on automatic and attention-free error detection and correction processes in children, as had been revealed in cursor-jump experiments in adults. To reach our goal, children and adults performed a video aiming task for which the cursor could be unpredictably and instantly translated by 20 mm. For the normal vision control trials or while the cursor was occluded approximately at midflight, we did not observe a significant difference in endpoint accuracy or movement time between children and adults. This result replicates previous observations (Pellizzer and Hauert, 1996; Mackrous and Proteau, 2010), when the participants aimed at a target located along their midline. Additionally, similar results were obtained for the normal vision and occluded conditions and were consistent with previous research showing that viewing the cursor on the starting base (Prablanc et al., 1979; Vindras et al., 1998; Bédard and Proteau, 2005) or briefly during movement execution (Bard et al., 1985; Abahnini and Proteau, 1999) might be used effectively for movement planning in subsequent trials. A new finding of the present study reveals that this information can also be used successfully for movement planning and control in young children. More importantly, in the context of the present study, these observations suggest that any differences in endpoint accuracy and variability across age groups for the cursor-jump trials resulted from differences in the processes responsible for detecting and correcting the cursor jump.

Previous research using a cursor-jump paradigm has revealed that the processes responsible for detecting and correcting target and cursor jumps are automatic or at least do not require attention in adult participants. Three lines of evidence have supported this position: (a) participants appropriately corrected their movement even when they were not consciously aware of a target (Prablanc et al., 1986) or cursor jump (Sarlegna et al., 2003, 2004; Saunders and Knill, 2003, 2004; Proteau et al., 2009), which occurred in the first perturbed trial (Brière and Proteau, 2011, 2016); (b) the detection and correction of a first cursor jump did not interfere with the detection and correction of a second cursor jump that occurred 100 ms after the initial jump (Brière and Proteau, 2011, 2016); and (c) when participants were instructed to move their hand in the same direction as the cursor jump or in the direction opposite of the target jump, they failed to refrain from correcting in the direction opposite to the cursor jump (Franklin and Wolpert, 2008) or in the same direction as the target jump (Day and Lyon, 2000; Pisella et al., 2000; Johnson et al., 2002).

As with previous studies (Sarlegna et al., 2003, 2004; Saunders and Knill, 2003, 2004; Proteau et al., 2009; Veyrat-Masson et al., 2010; Brière and Proteau, 2011), debriefed participants in the present study reported that they were not aware that the cursor had jumped on some trials. Nonetheless, participants in all age groups amended their movements to counteract the cursor jump. These corrections did not differ significantly between age groups when the cursor jumped to the right (average of 6.5 mm or 33% of the perturbation). However, when the cursor jumped to the left, the oldest children produced larger corrections than the two younger groups, and all children committed larger corrections than the adults. A likely explanation for this finding is that the correction for a cursor jump to the right required that the participants’ right hand cross their midline to reach the target, which was not the case when the cursor jumped to the left. This results in biomechanical constraints affecting movement velocity and accuracy (Carey et al., 1996; see also Trempe and Proteau, 2008). Nonetheless, because corrections were observed for both right and left cursor jumps, we can conclude that the automatic online error detection and correction processes revealed in previous cursor-jump studies also occur in young children.

However, as for the correction for a target jump (Hyde and Wilson, 2013; Wilson and Hyde, 2013), it appears that these processes are more efficient in older than in younger children. This advantage of age does not strongly appear for any dependent variable, but it occurs when the results of different dependent variables are considered simultaneously, thus demonstrating a developmental trend. Specifically, we observed that the oldest group of children initiated their corrections with a shorter latency than the youngest group. The oldest group of children also had shorter and less variable movement times but showed larger and less variable corrections for the cursor jump. These more efficient corrections for cursor jumps in the oldest group of children might have resulted from a better estimation of the size of the jump and/or planning of a more efficient correction. However, our results cannot answer this important question.

Notably, if the correction for the cursor jump had been voluntary in children, it would have occurred during the end phase of the movement (between peak deceleration and the movement endpoint), as observed in previous research (Lhuisset and Proteau, 2004; Wilson and Hyde, 2013). Therefore, we should not have observed efficient corrections for the left jump trials for which cursor visibility was occluded at approximately midflight. Instead, our results suggested that the initial impulse of goal-directed movements was continuously monitored by visual feedback, even in the youngest group of children. Thus, this monitoring and the resulting correction were relatively automatic and did not require many resources.

In the present study, we observed that a relatively high proportion of the trials performed by children showed a secondary corrective impulse. The ability to perform an efficient secondary correction, even in the absence of visual feedback (left jump, occluded), required the children to use information from the initial movement impulse, the final viewed position of the cursor and the effect of the automatic correction that had been or would be initiated to predict where this correction would lead the cursor relative to the target and then to use this information to plan a secondary correction. This result indicates that the children’s movements were controlled by a forward process, although, underdeveloped in the youngest children. Therefore, our results have more resemblance than dissemblance with those reported in target jump studies (Hyde and Wilson, 2013; Wilson and Hyde, 2013). In turn, this suggests that the dedicated channel proposed by Reichenbach et al. (2014) for the processing of visual hand information develops during childhood.

Three aspects of our data require further consideration. First, we observed larger corrections for the cursor jump in children than adults, which was unexpected. Second, the 8- to 9-years-old group had longer movement times than all other age groups. Third, the participants did not fully correct their movements for the cursor jump. Concerning this latter point, less complete corrections typically occur in cursor-jump experiments (see Sarlegna et al., 2003, 2004; Saunders and Knill, 2003, 2004; Proteau et al., 2009; Sarlegna and Blouin, 2010; Brière and Proteau, 2016 for similar results). These partial corrections might result because the cursor jump creates a dissociation between the felt and observed position of one’s hand. The possible conflict resulting from this dissociation might limit the size of the correction (Sarlegna et al., 2004; Brière and Proteau, 2016). In addition, for a given cursor jump, the size of the corrections observed in different studies depends on many procedural differences. For instance, Proteau and colleagues (Proteau et al., 2009; Veyrat-Masson et al., 2010; Brière and Proteau, 2011, 2016) used an apparatus similar to ours and observed corrections that accounted for 65–85% of the perturbation. However, in this previous study, the target was located between 20 and 32 cm from the starting base, whereas to accommodate the arm’s length of our youngest group of children in the present study, the target was located 15 cm from the starting base. Additionally, participants in the previous study completed their movements in 800 ms (± 15%), whereas in the present study, the prescribed movement time was 500 ms (± 12%).

The shorter movements and shorter movement times used in the present study compared to the previously cited study most likely explain why we observed smaller cursor-jump corrections. The shorter movement time frame when introducing the cursor jump after movement initiation obviously decreased the time available to perform the correction. Therefore, it was not surprising that we observed smaller corrections in the present study compared to previous research (see also Saunders and Knill, 2003 for supporting evidence). Additionally, the correction does not occur instantaneously once the jump is detected and instead occurs gradually. Therefore, in addition to the short correction time frame, the participants did not have much space to perform the correction, which was consistent with our smaller correction observations compared to the findings of Proteau et al. (2009). However, why were children more proficient in correcting for a left cursor jump than adults?

The smaller cursor-jump corrections by adults than by children was an unexpected result. However, we also noted that adults complied better than children with the imposed movement time (smaller movement time variability) and our request to complete their movement in a single motion. In our opinion, because of the relatively short movement time used in the present study, this variable might have resulted in adults terminating their movement prior to the completion of the cursor-jump correction. This result is consistent with Liu and Todorov (2007) who showed that the size of the target jump correction was influenced by the stabilization requirement of the task: the need for a greater endpoint stability resulted in a smaller target jump correction. The relative inability of the children to refrain from performing a secondary correction and because they had difficulty complying with the imposed movement time suggested that children might use a different stabilization-accuracy trade-off than adults. Children might be more sensitive to positional than temporal errors, potentially because they are easier to detect.

Finally, the longer movement times observer for the 8- to 9-years-old group add to a rather large body of data (for example, Hay, 1978, 1979; Bard et al., 1990; Fayt et al., 1992; Pellizzer and Hauert, 1996; Lhuisset and Proteau, 2004) indicating that the behavior of 8-years-old children differs markedly from that of both younger and older children. That finding might indicate that children of age 8 are going through some important modifications in how they process information for movement planning and control.

## Conclusion

The results of the present study obviously show that the initial portion of goal-directed movements were monitored visually, even in young children. This monitoring and the error correction process that it triggers appears to be automatic because it occurs even when the cursor jump, which we introduced in some trials, was not detected consciously. Finally, this process was not fully developed before late childhood, which suggests that the dedicated channel proposed by Reichenbach et al. (2014) for the processing of visual hand information develops during childhood.

## Author Contributions

LP and IM: designed the experiment. IM: was responsible for data collection. LP and IM: analyzed the data. LP and IM: wrote the final version of the paper.

## Conflict of Interest Statement

The authors declare that the research was conducted in the absence of any commercial or financial relationships that could be construed as a potential conflict of interest.
